# Dual-phase CT radiomics for acute kidney injury prediction after out-of-hospital cardiac arrest

**DOI:** 10.3389/fradi.2026.1875412

**Published:** 2026-06-26

**Authors:** Michael Scheschenja, Lisa Hekers, Julian Kreutz, Birgit Markus, Susanne Betz, Jarmila Jedelská, Alexander König, Andreas H. Mahnken, Simon Viniol

**Affiliations:** 1 Diagnostic and Interventional Radiology, Marburg University Hospital, Philipps-University Marburg, Marburg, Germany; 2 Cardiology, Angiology, and Intensive Care Medicine, Marburg University Hospital, Philipps-University Marburg, Marburg, Germany; 3 Center for Emergency Medicine, Marburg University Hospital, Philipps-University Marburg, Marburg, Germany; 4 Diagnostic and Interventional Radiology and Nuclear Medicine, St. Josef-Hospital, University Hospital Bochum, Bochum, Germany

**Keywords:** acute kidney injury, cardiac arrest, computed tomography, machine learning, radiomics, return of spontaneous circulation

## Abstract

**Background:**

Acute kidney injury (AKI) is common and prognostically relevant after out-of-hospital cardiac arrest (OHCA), yet early risk prediction at admission remains limited.

**Objective:**

To assess whether radiomics features from dual-phase contrast-enhanced CT obtained at admission predict AKI after non-traumatic OHCA.

**Methods:**

This retrospective single-center study included consecutive non-traumatic OHCA patients with return of spontaneous circulation undergoing standardized admission dual-phase CT. AKI within 5 days was defined by KDIGO criteria or renal replacement therapy. Bilateral whole-kidney masks were generated using TotalSegmentator, reviewed, and corrected if necessary. Radiomics features were extracted with PyRadiomics within 3DSlicer. Features with poor reproducibility (CCC < 0.75) were excluded. Arterial-venous difference features were computed. Data were split into a training cohort and test cohort. Feature selection used mRMR followed by LASSO regression, yielding seven predictors for training of a logistic regression (LR) model, a support vector machine (SVM), and a k-nearest neighbors (kNN) model. Additionally, a limited clinical variable LR model and a combined model were evaluated. Stability was assessed by *post hoc* repeated resampling of the primary radiomics LR model across 100 stratified 70/30 splits, summarizing repetitions yielding 3–9 LASSO-selected features.

**Results:**

Of 383 screened patients, 155 were included, of which 47 (30.3%) developed AKI. In the test cohort, radiomics-LR showed the highest AUC (0.783), with kNN (0.778) and SVM (0.757) performing comparable. The limited clinical model performed poorly (AUC 0.549), and the combined model (AUC 0.779) did not materially improve upon radiomics alone. In the repeated resampling analysis, 29 of 100 repetitions fulfilled the predefined feature-count criterion. Among these repetitions, median test-set AUC was 0.663 (IQR, 0.623–0.699), indicating moderate but variable discriminatory performance and limited feature-selection stability. Most selected predictors were arterial-venous difference features.

**Conclusions:**

This exploratory single-center proof-of-concept study suggests that admission dual-phase CT radiomics may contain information relevant to early AKI risk stratification after OHCA, with arterial-venous difference features appearing particularly informative. However, limited stability of feature selection and model performance indicates that these findings should be considered hypothesis-generating and require methodological refinement, comparison with more comprehensive clinical models, and external validation in prospective multicenter cohorts before clinical application can be considered.

## 1 Introduction

Out-of-hospital cardiac arrest (OHCA) remains a major global health challenge with persistently high mortality rates (1). Among survivors of OHCA, systemic ischemia-reperfusion injury frequently results in multi-organ dysfunction, with acute kidney injury (AKI) being a common and clinically significant complication. The most recent meta-analysis reported an incidence of approximately 40% among CA survivors (2). Post-CA AKI is independently associated with prolonged hospitalization, poor neurological outcomes, and, most importantly, increased mortality (2–4).

Despite these well-documented consequences, accurate early prediction of AKI in the immediate post-resuscitation period has received relatively little attention. Current predictors and predictive approaches rely primarily on clinical variables and laboratory biomarkers, which remain limited in their discriminative capacity and leave substantial room for improvement (5–8). This underscores the need for novel, multimodal strategies capable of identifying patients at high risk for AKI early after admission, thereby enabling timely intervention and potentially improving outcomes.

One way to enhance prediction with imaging information is through radiomics and machine learning-based modeling. Radiomics augments outcome prediction by adding quantitative information from routine imaging to machine learning models. It extracts large sets of image features that capture tissue heterogeneity, texture, and morphology beyond visual assessment (9–11). Over recent years, radiomics has been successfully applied across diverse domains, also including studies in the critical care setting (12–16). Beyond that, there is evidence supporting the utility of radiomics and other imaging-based predictive approaches specifically in the context of kidney injury and kidney function decline (13, 17–19). Notably, contrast-enhanced CT is frequently acquired at admission after OHCA and dual-phase protocols offer arterial and venous information from which different features can be derived. These may reflect perfusion and microvascular alterations relevant to early renal injury.

Nevertheless, no study to date has specifically evaluated the role of radiomics for AKI prediction in patients following OHCA. This represents a clinically significant knowledge gap, as early identification of renal complications in this vulnerable cohort may guide closer monitoring, timely initiation of nephroprotective measures, and individualized therapy.

This study aimed to generate preliminary evidence on whether radiomics features extracted from contrast-enhanced CT images acquired immediately after hospital admission can predict the occurrence of AKI in patients with OHCA following return of spontaneous circulation (ROSC).

## 2 Materials and methods

### 2.1 Study design

The local institutional ethics committee approved this retrospective study and the need for informed consent was waived. CLEAR-S- and METRICS-Checklists were used for reporting and are available as Supplementary Material (11, 20). Patients were consecutively included between 01.06.2016 and 31.12.2023 if they met the following criteria: (1) age > 18 years (2) documented non-traumatic OHCA with subsequent ROSC; and (3) availability of contrast-enhanced computed tomography (CT) of the abdomen as part of a whole-body post-OHCA CT protocol at hospital admission, acquired according to the institutional standard protocol.

For each eligible patient, clinical data related to the OHCA event (including demographic information, initial cardiac rhythm, time to ROSC, and laboratory parameters at admission) were collected. Serum creatinine values were collected during the first five days of hospitalization.

Exclusion criteria were: (1) pre-existing severe renal dysfunction, defined as an estimated glomerular filtration rate (eGFR) < 30 mL/min/1.73 m^2^ at admission; (2) missing or insufficient clinical or imaging data; or (3) death within the first five days after hospital admission.

Given the retrospective exploratory design, the final sample size was determined based on the availability of consecutive eligible patients during the predefined study period. The study cohort was derived from the same institutional post-OHCA imaging database as a previous study (15). Therefore, partial patient overlap is possible. However, study-specific data extraction and analysis were performed independently.

AKI was defined by a rise of serum creatinine values according to the Kidney Disease: Improving Global Outcomes (KDIGO) criteria (21) within the first five days after admission, with admission creatinine serving as baseline, or by the initiation of renal replacement therapy during the same five-day period.

### 2.2 Image acquisition and reconstruction

All included patients underwent whole-body contrast-enhanced CT immediately upon hospital admission, as part of the standardized institutional post-OHCA protocol. Imaging was performed on a Somatom Definition AS scanner (Siemens Healthineers, Erlangen, Germany) using a biphasic acquisition protocol, which included both an early arterial and a venous phase.

A total of 120 mL of contrast medium (Ultravist 370, Bayer, Leverkusen, Germany) was administered intravenously via a dual-syringe injector pump (Stellant D CT Injection System, MEDRAD; Bayer, Leverkusen, Germany) using the following protocol: First, 80 mL of contrast medium followed by an additional 40 mL of contrast medium mixed with 40 mL of 0.9% saline solution, and finally by a 50 mL 0.9% saline chaser bolus. Flow rate was 4 mL/s for all injections. For contrast timing, bolus tracking was applied at the thoracic aorta with a predefined threshold of 140 Hounsfield units (HU); the arterial phase scan commenced 2 s after the threshold was reached. The venous phase acquisition was initiated with a 30 s delay after completion of the arterial phase.

During the arterial phase acquisition, scanning was performed with a tube voltage of 120 kV, tube current modulation using automatic exposure control (CARE Dose4D), detector collimation of 64 × 0.6 mm, a pitch of 0.85 and craniocaudal scan direction. During the venous phase acquisition, scanning was performed with a tube voltage of 120 kV, tube current modulation using automatic exposure control (CARE Dose4D), detector collimation of 64 × 0.6 mm, a pitch of 0.6 and craniocaudal scan direction.

For the arterial phase, images were reconstructed with a slice thickness of 2 mm, an increment of 1.5 mm, and a B30f kernel. For the venous phase, reconstruction was performed with a slice thickness of 3 mm, an increment of 2 mm, and a B31f kernel.

### 2.3 Segmentation and radiomics feature extraction

**Figure 1:**
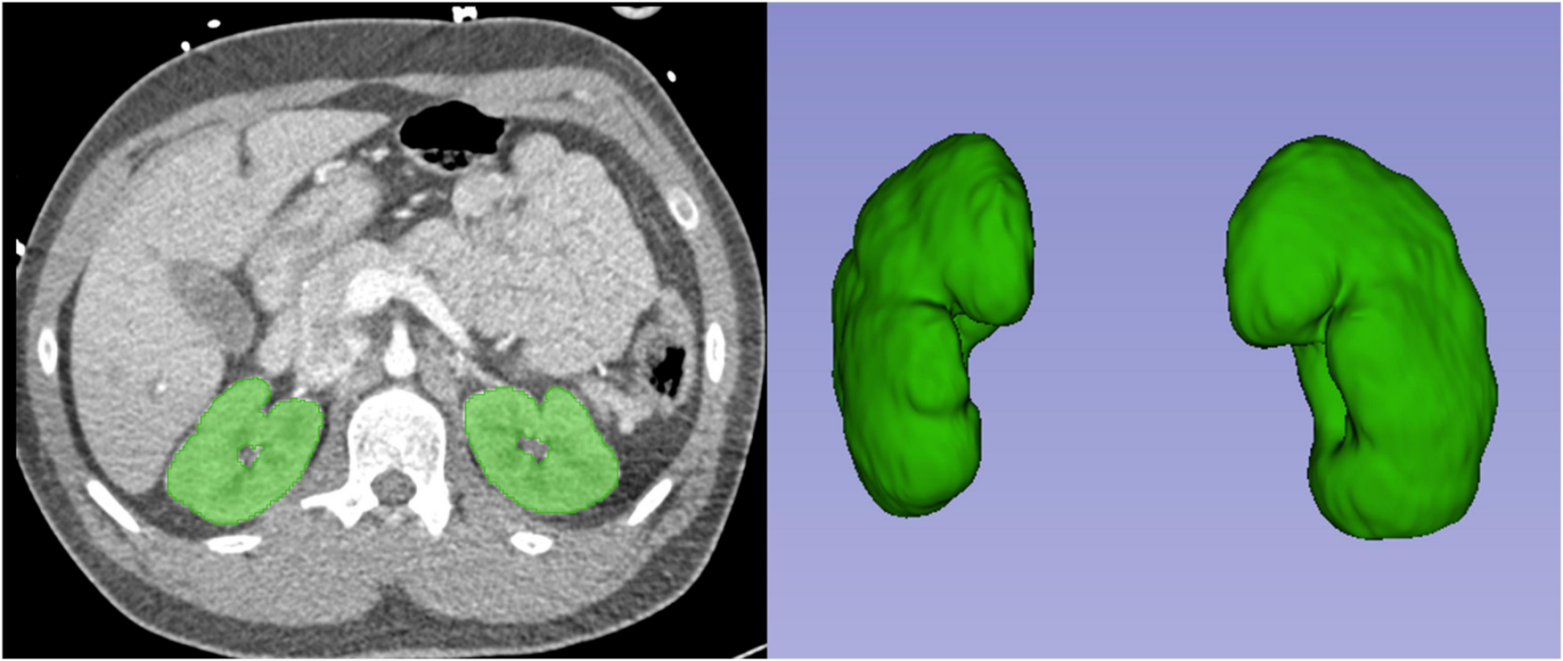
Example of kidney segmentation and 3D rendering. Axial contrast-enhanced CT slice at admission with bilateral kidney masks and 3D surface rendering of both kidneys derived from the same segmentation.

All CT images were exported in Digital Imaging and Communications in Medicine (DICOM) format for further processing. Kidney segmentation was performed using 3D Slicer (v5.2.2, http://www.slicer.org) in combination with the TotalSegmentator extension (22). TotalSegmentator was used to generate initial bilateral whole-kidney segmentation masks separately on arterial and venous phase CT images. No image registration between arterial and venous phases was performed. The resulting masks were visually reviewed for anatomical plausibility and, if necessary, manually corrected by a trained final-year medical student completing a radiology rotation who was blinded to AKI outcome. Prior to segmentation, the observer was trained using representative example cases and performed the segmentation workflow under supervision of a fourth-year radiology resident. The observer could consult the supervising radiologist in cases of uncertainty. A representative kidney segmentation is shown in Figure 1.

Radiomics feature extraction was carried out using the PyRadiomics (version 3.0.1) module integrated within 3D Slicer. Before feature extraction, all images were resampled to an isotropic voxel size of 1 × 1 × 1 mm, and the gray-level discretization bin width was set to 25. All other parameters remained at their default settings. No additional thresholding, intensity capping, or image-intensity normalization was applied prior to feature extraction. This process yielded a total of 837 radiomics features per region of interest (ROI), covering multiple feature classes: first-order statistics, gray-level co-occurrence matrix (GLCM), gray-level dependence matrix (GLDM), gray-level run length matrix (GLRLM), gray-level size zone matrix (GLSZM), neighboring gray-tone difference matrix (NGTDM), and wavelet-transformed features.

### 2.4 Feature selection and predictive modeling

Radiomics features were preprocessed prior to model development by excluding non-reproducible variables, defined as those with a concordance correlation coefficient (CCC) below 0.75 based on the repeated segmentations of a random subset of 40 CT scans. All remaining features were normalized using a min-max scaling approach. This was performed as an unsupervised preprocessing step on the full dataset and did not use AKI outcome information. For each feature available in both arterial and venous contrast-enhanced phases, an arterial-venous difference feature was computed at the feature level as arterial value minus venous value to capture phase-dependent contrast information.

The dataset was randomly split into a training cohort (70%) and a separate test cohort (30%) using stratified sampling to preserve the proportion of AKI outcomes and a fixed random seed (123). All subsequent feature selection and model-development steps were exclusively performed within the training cohort. Feature selection was conducted using the minimum redundancy maximum relevance (mRMR) algorithm to identify the 100 most relevant and least redundant features with respect to the target variable. Subsequently, a least absolute shrinkage and selection operator (LASSO) regression with five-fold cross-validation was applied to this reduced feature set. The final subset of features corresponding to the one-standard-error (*λ*₁ₛₑ) penalty parameter was retained for model development.

Three radiomics-based classifiers were trained on the selected features exclusively within the training cohort: logistic regression (LR), support vector machine (SVM) with radial basis function kernel, and k-nearest neighbors (kNN) classifier. For the kNN model, the optimal number of neighbors (k) was determined via repeated five-fold cross-validation within the training dataset, optimizing the mean AUC. The final k value was then used to train the kNN model on the entire training set.

Potential clinical pre- and intra-arrest confounding variables were considered during model development and interpretation. Based on prior literature and clinical plausibility, age, sex, initial cardiac rhythm, time to ROSC, and admission creatinine were considered clinically relevant covariates potentially associated with AKI risk (5). These variables were therefore included in a limited clinical LR-model and in a combined LR-model using both the selected radiomics features and clinical variables to assess whether radiomics features provided information beyond selected admission clinical parameters. These models were also exclusively trained within the training cohort and evaluated using the test cohort.

For reporting general model performance in the relatively balanced dataset, sensitivity, specificity, F1 score and AUC were calculated for all approaches in both training and separate test cohorts with receiver operating characteristic (ROC) curves generated for visualization. Sensitivity, specificity, and F1 score were calculated using a fixed probability threshold of 0.5. For the radiomics-only LR, the limited clinical LR, and the combined (radiomics + clinical) LR models, test-set AUCs were compared pairwise using DeLong's test with *α* = 0.05.

Between-group comparisons of baseline characteristics (AKI vs. no AKI) used the Wilcoxon rank-sum test for continuous variables (age, time to ROSC, admission creatinine) and the chi-square test for categorical variables (sex, initial rhythm) with *α* = 0.05.

**Figure 2:**
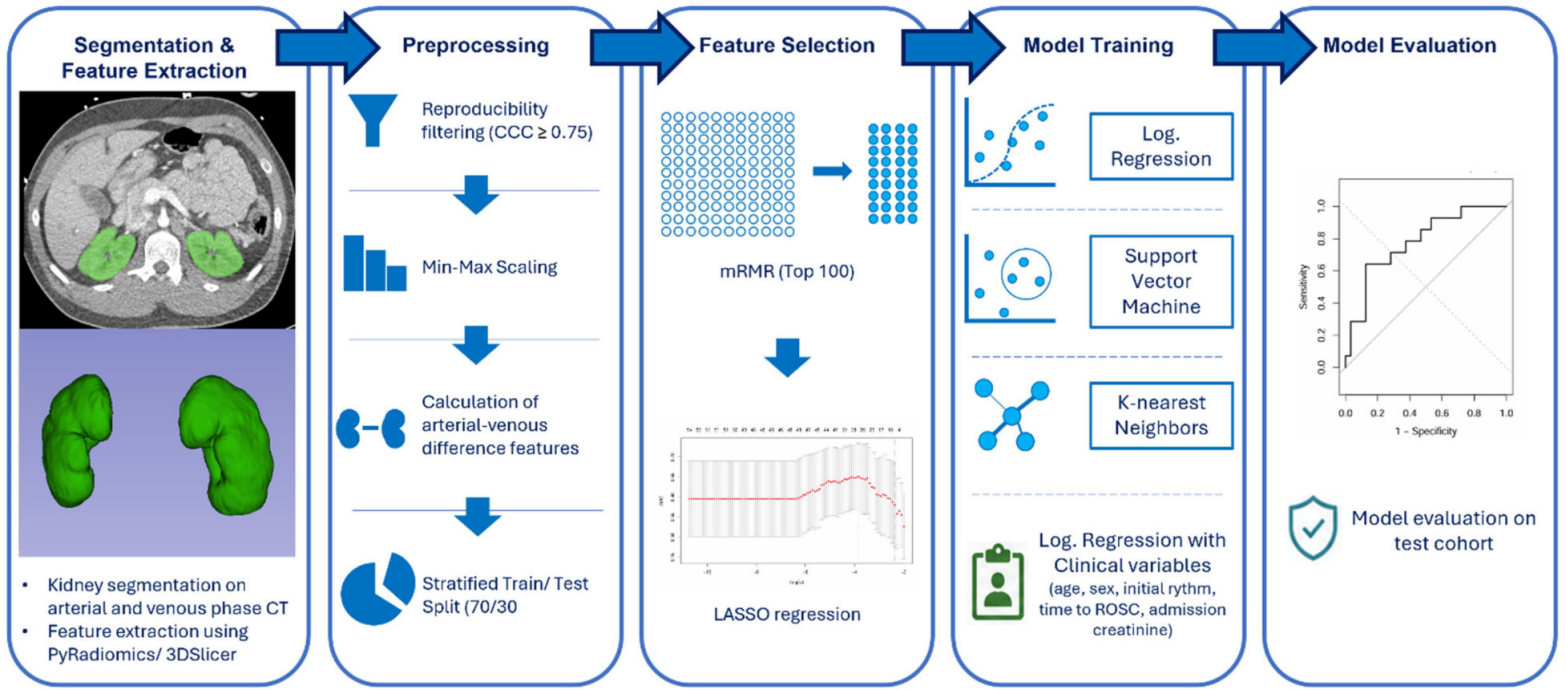
Radiomics workflow for prediction of acute kidney injury after out-of-hospital cardiac arrest. Kidneys were segmented on arterial and venous phase contrast-enhanced CT images, followed by radiomics feature extraction. Features underwent reproducibility filtering using a concordance correlation coefficient threshold of ≥ 0.75, min-max scaling, and calculation of arterial–venous difference features. The dataset was split into stratified training and test cohorts (70/30). Feature selection was performed using mRMR followed by LASSO regression. Radiomics-based logistic regression, support vector machine, and k-nearest neighbors models were trained in the training cohort and evaluated in the internal test cohort. Additional clinical models were trained using age, sex, initial rhythm, time to ROSC, and admission creatinine.

The complete radiomics workflow is summarized in Figure 2.

To further assess the stability of model performance and feature selection, a *post hoc* repeated resampling analysis was performed for the primary radiomics logistic regression model. The modeling pipeline, including mRMR feature preselection, LASSO regression, model training, and test-set evaluation, was repeated across 100 random stratified 70/30 train-test splits as described above. Given the exploratory aim and to allow performance estimation for models with appropriate feature-set sizes, performance was summarized for repetitions in which LASSO selected a comparable predefined feature-set size of 3–9 features. Repetitions outside this feature-count range were not used for performance estimation. Test-set performance was summarized using median AUC with interquartile range, and selected feature sets were analyzed to assess feature-selection frequencies.

All analyses were performed using R version 4.2.2 (R Foundation for Statistical Computing, Vienna, Austria) and Excel (Microsoft, Redmond, WA, USA).

## 3 Results

### 3.1 Study cohort

**Figure 3:**
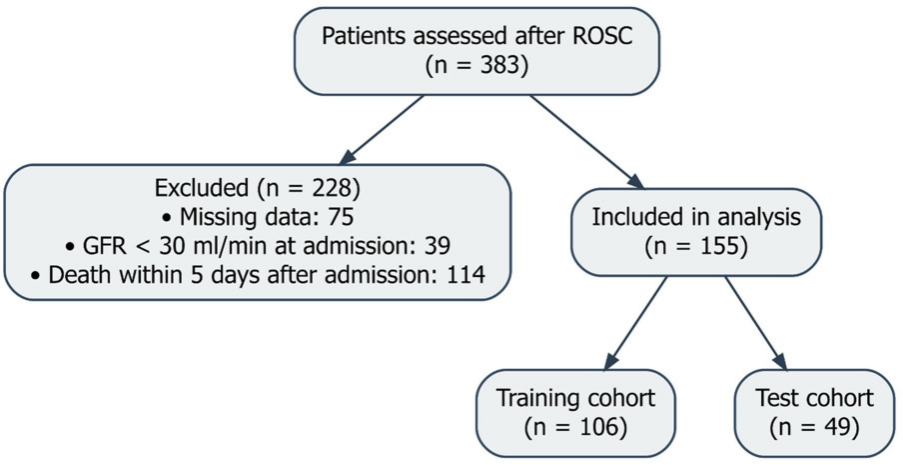
Patient selection flowchart. A total of 383 patients were screened after OHCA with ROSC. Exclusions: missing clinical/imaging data (*n* = 75), pre-existing severe renal dysfunction (eGFR < 30 mL/min/1.73 m^2^; *n* = 39), and death within 5 days after admission (*n* = 114). The final study cohort comprised 155 patients.

Between June 2016 and December 2023, a total of 383 patients were screened for eligibility. After exclusion of patients with pre-existing severe renal dysfunction (estimated glomerular filtration rate < 30 mL/min/1.73 m^2^), missing clinical or imaging data, or death within the first five days after admission, 155 patients were included in the final analysis (Figure 3).

The baseline characteristics of the study cohort are summarized in Table 1. The mean age was 70.3 ± 13.3 years, and most patients were male (74.2%). The mean time to ROSC was 19.1 ± 11.5 min. The mean serum creatinine concentration at admission was 1.21 ± 0.33 mg/dL. Initial cardiac rhythm was ventricular fibrillation/flutter in 51.0%, asystole in 25.2%, and pulseless electrical activity in 23.9% of cases. Within the first five days after admission, 47 patients (30.3%) developed AKI.

**Table 1:** Baseline characteristics of the study cohort with cardiac arrest and return of spontaneous circulation (ROSC). Acute kidney injury (AKI) was defined by KDIGO criteria or initiation of renal replacement therapy.

Age [years]	Sex distribution	Time to ROSC [mins]	Creatinine at admission [mg/dL]	Initial rhythm	Number of AKI after admission
70.3 (±13.3)	Male = 115 (74.2%) Female = 40 (25.8%)	19.1 (±11.5)	1.21 (±0.33)	Ventricular fibrillation/flutte*r* = 79 (51%) Asystole = 39 (25.2%) Pulseless electrical activity = 37 (23.9%)	47 (30.3%)

Baseline characteristics stratified by AKI status are summarized in Table 2. Among patients without AKI (*n* = 108), the mean age was 70.2 ± 13.1 years, 75.0% were male, the mean time to ROSC was 18.6 ± 11.6 min, the mean admission serum creatinine was 1.17 ± 0.30 mg/dL and the initial rhythm was ventricular fibrillation/flutter in 56.5%, asystole in 21.3%, and pulseless electrical activity in 22.2% of cases. Among patients with AKI (*n* = 47), the mean age was 70.4 ± 14.0 years, 72.3% were male, the mean time to ROSC was 20.5 ± 11.4 min, the mean admission serum creatinine was 1.30 ± 0.37 mg/dL and the initial rhythm was ventricular fibrillation/flutter in 38.3%, asystole in 34.0%, and pulseless electrical activity in 27.7% of cases.

**Table 2:** Characteristics of the study cohort with out-of-hospital cardiac arrest and return of spontaneous circulation (ROSC), stratified by acute kidney injury (AKI) status. Continuous variables are summarized as mean (SD) and compared using the Wilcoxon rank-sum test. Categorical variables are shown as *n* (%) and compared using the chi-square test.

Parameter	Patients without AKI (*n* = 108)	Patients with AKI (*n* = 47)	*p*-value
Age [years]	70.2 (±13.1)	70.4 (±14.0)	0.904
Sex distribution	Male = 81 (75.0%) Female = 27 (25.0%)	Male = 34 (72.3%) Female = 13 (27.7%)	0.728
Time to ROSC [mins]	18.6 (±11.6)	20.5 (±11.4)	0.197
Creatinine at admission [mg/dL]	1.17 (±0.30)	1.30 (±0.37)	0.004
Initial rhythm	Ventricular fibrillation/flutte*r* = 61 (56.5%) Asystole = 23 (21.3%) Pulseless electrical activity = 24 (22.2%)	Ventricular fibrillation/flutter =18 (38.3%) Asystole = 16 (34.0%) Pulseless electrical activity = 13 (27.7%)	0.098

The training cohort comprised 109 patients, including 33 patients with AKI, whereas the internal test cohort comprised 46 patients, including 14 patients with AKI. Baseline characteristics within training cohort and test cohort are included in the Supplementary Material.

**Figure 4:**
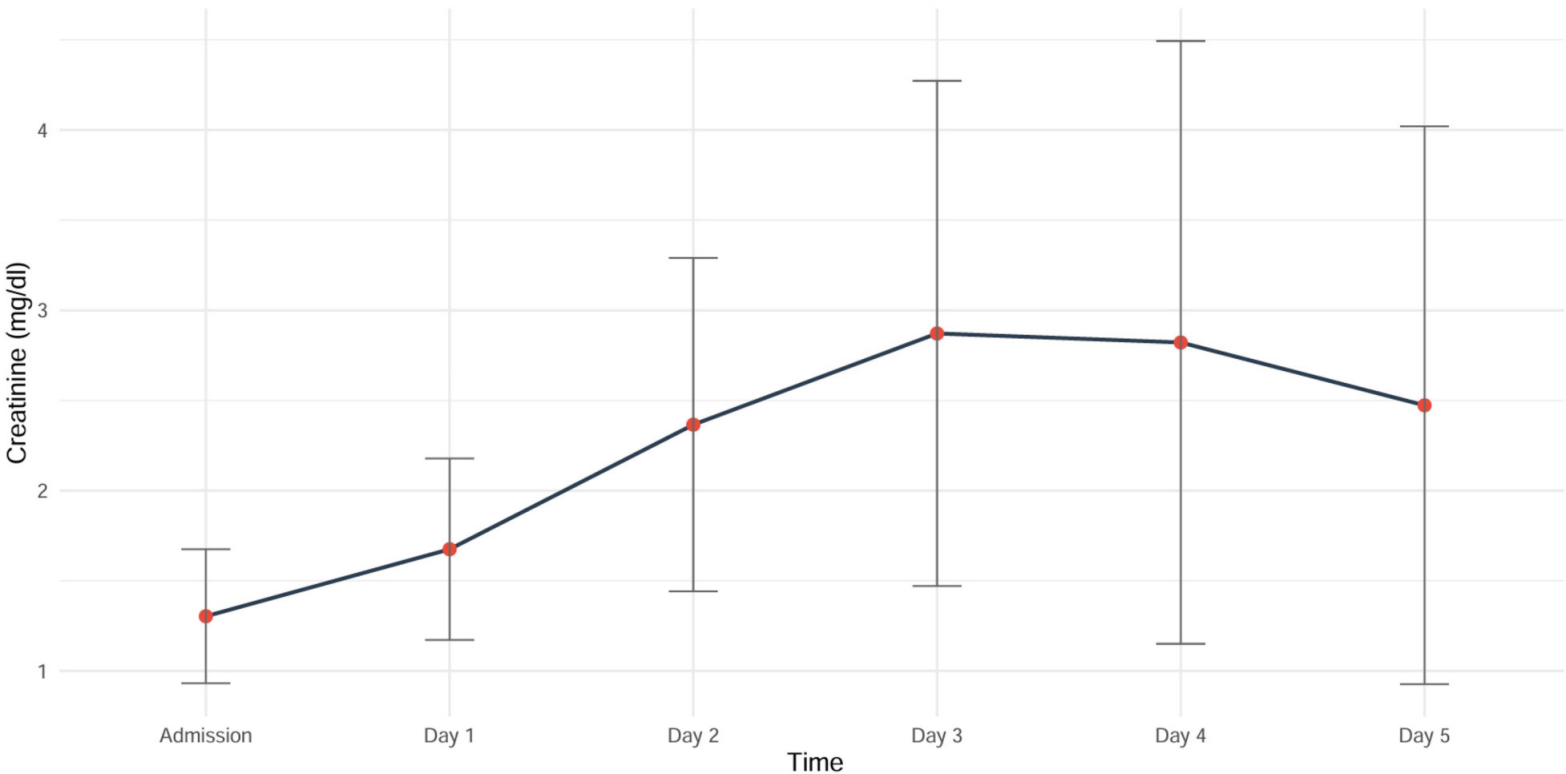
Trajectory of serum creatinine during the first 5 days after admission among patients who developed AKI (*n* = 47). Mean (±SD) creatinine values at admission and days 1−5 are shown. Values peak around post-arrest day 3 and decline thereafter.

Longitudinal creatinine levels of patients with AKI are summarized in Table 3 and visualized in Figure 4, showing a peak around day 3 followed by a gradual decline thereafter.

**Table 3:** Serum creatinine during the first 5 days after admission among patients who developed AKI (*n* = 47).

Time point	Mean (SD), mg/dL
Admission	1.30 (±0.37)
Day 1	1.67 (±0.50)
Day 2	2.37 (±0.92)
Day 3	2.87 (±1.40)
Day 4	2.82 (±1.67)
Day 5	2.47 (±1.55)

### 3.2 Radiomics feature selection

A total of 837 radiomics features were extracted per contrast phase. After reproducibility screening (CCC ≥ 0.75), 365 features remained in the arterial phase and 350 in the venous phase. For features available in both phases, 290 arterial-venous difference features (arterial minus venous) were computed. Using the mRMR algorithm, the total feature count was reduced to 100 variables. The final features for model building retained by LASSO regression were:

“arterial_wavelet_HLL_glrlm_RunEntropy”
  “diff_wavelet_LLL_firstorder_10Percentile”
  “diff_wavelet_HLL_firstorder_Skewness”
  “diff_wavelet_LLL_glszm_GrayLevelNonUniformity”
  “diff_wavelet_LLL_gldm_LargeDependenceHighGrayLevelEmphasis”
  “diff_wavelet_LLL_glszm_LargeAreaHighGrayLevelEmphasis”
  “arterial_original_glcm_MCC”

Analysis of feature data is included in the Supplementary Material.

### 3.3 Model performance

**Figure 5:**
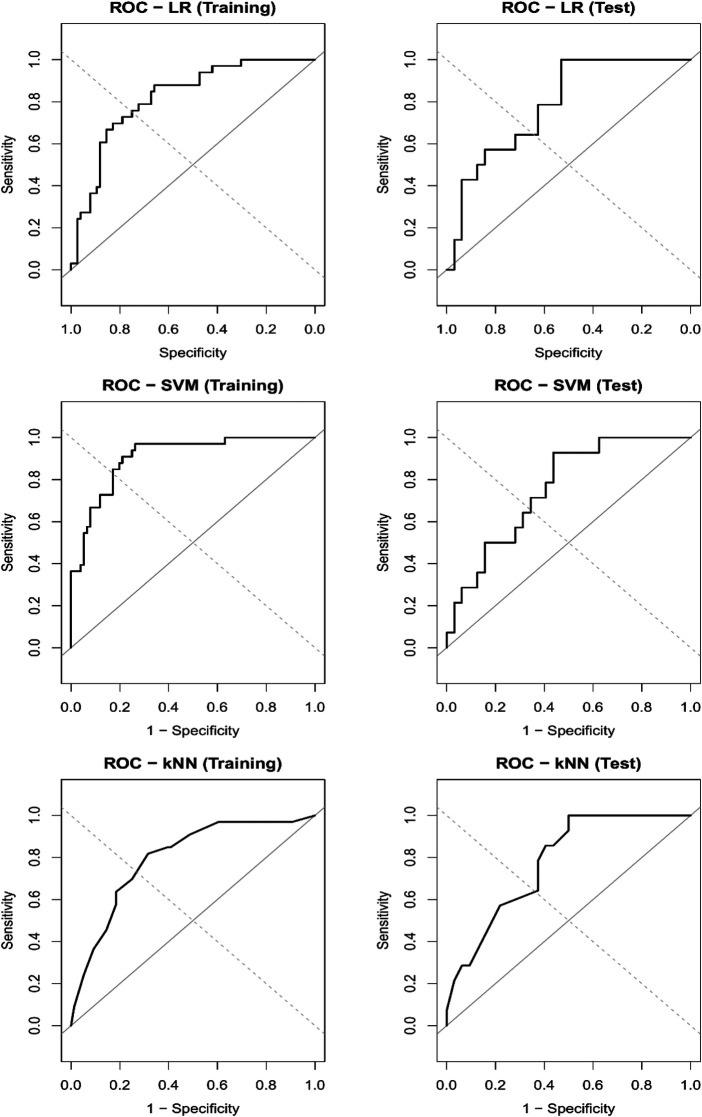
Receiver operating characteristic (ROC) curves of radiomics-only models in training and test cohorts. Logistic regression (LR), support vector machine (SVM), and k-nearest neighbors (kNN) trained on selected radiomics features.

Performance metrics with 95% confidence intervals of the three radiomics-based classifiers (LR, SVM and kNN) are reported in Table 4 and visualized in Figure 5. Across the training cohort, the SVM achieved the highest AUC (0.907), followed by LR (0.821) and kNN (0.796). In the separate test cohort, LR with radiomics features showed the highest test-set AUC (AUC 0.783), followed closely by kNN (AUC 0.778), while the SVM exhibited somewhat lower generalizability (AUC 0.757).

**Table 4:** Performance metrics of radiomics-only models in training and test cohorts reported with 95% confidence intervals.

Model	Cohort	Sensitivity (%)	Specificity (%)	AUC (95% CI)	F1 score (95% CI)
Logistic regression	Training	45.5 (28.1–63.6)	88.2 (78.7–94.4)	0.821 (0.739–0.902)	0.526 (0.353–0.676)
	Test	57.1 (28.9–82.3)	84.4 (67.2–94.7)	0.783 (0.649–0.918)	0.593 (0.348–0.788)
Support vector machine	Training	54.5 (36.4–71.9)	94.7 (87.1–98.5)	0.907 (0.850–0.964)	0.655 (0.485–0.788)
	Test	35.7 (12.8–64.9)	84.4 (67.2–94.7)	0.757 (0.615–0.898)	0.417 (0.143–0.643)
K-nearest neighbors	Training	45.5 (28.1–63.6)	85.5 (75.6–92.5)	0.796 (0.708–0.885)	0.508 (0.333–0.657)
	Test	57.1 (28.9–82.3)	78.1 (60.0–90.7)	0.778 (0.645–0.911)	0.552 (0.300–0.750)

**Figure 6:**
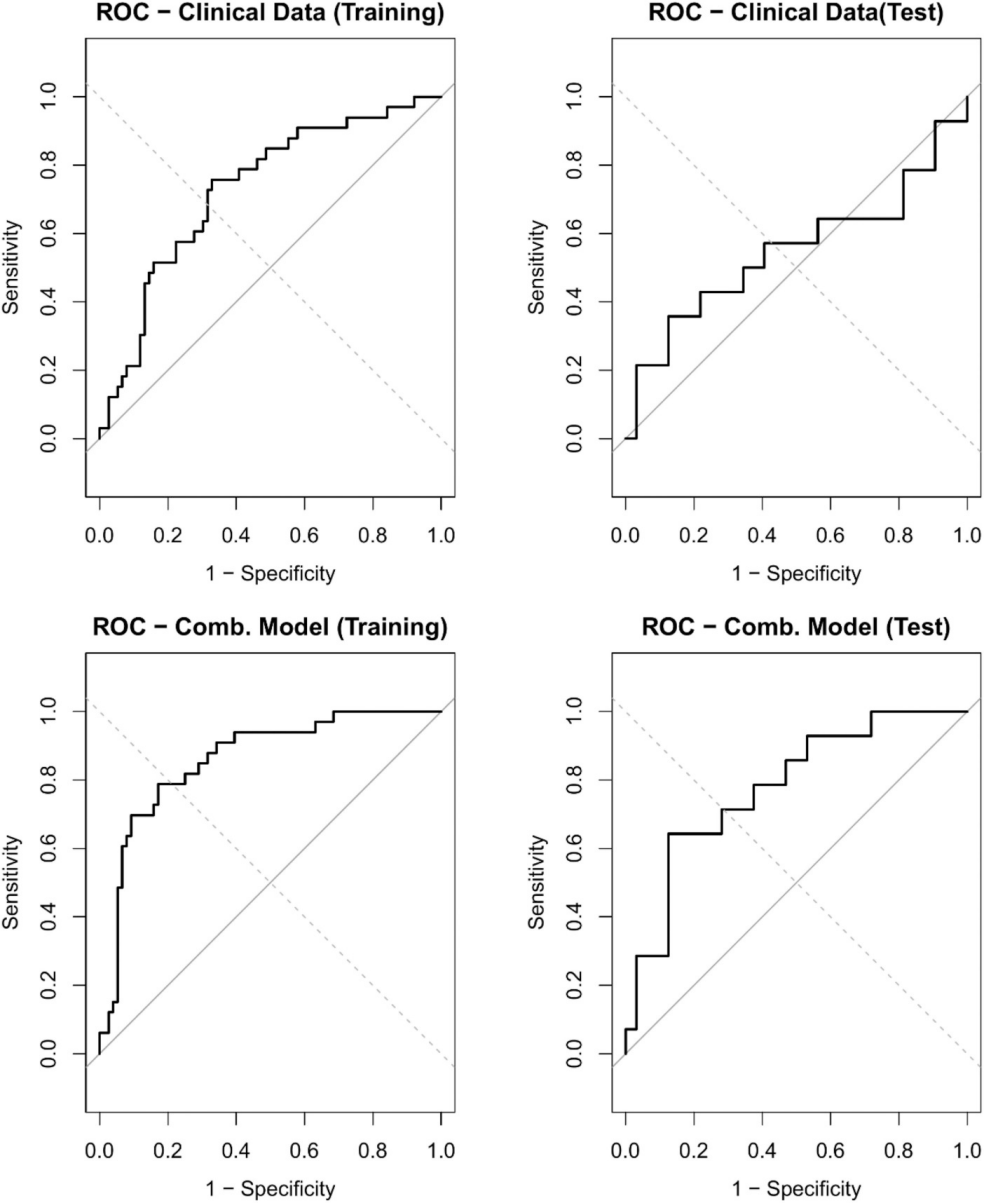
ROC curves of clinical and combined (clinical + radiomics) models in training and test cohorts. Clinical model (age, sex, initial rhythm, time to ROSC, admission creatinine) versus combined model integrating the same clinical variables with the selected radiomics features.

The LR model trained on clinical data alone demonstrated limited predictive ability, with an AUC of 0.731 in the training cohort and 0.549 in the test cohort. The combined model integrating radiomics and clinical features achieved an AUC of 0.860 in the training set and 0.779 in the test set, comparable to the performance of the radiomics-only LR model (Table 5, Figure 6).

**Table 5:** Performance metrics of the clinical and combined (clinical + radiomics) models in training and test cohorts reported with 95% confidence intervals.

Model	Cohort	Sensitivity (%)	Specificity (%)	AUC (95% CI)	F1 score (95% CI)
Logistic regression with clinical data	Training	21.2 (9.0–38.9)	89.5 (80.3–95.3)	0.731 (0.630–0.833)	0.292 (0.121–0.459)
	Test	35.7 (12.8–64.9)	78.1 (60.0–90.7)	0.549 (0.340–0.758)	0.385 (0.133–0.611)
Logistic regression with clinical data and radiomics features	Training	60.6 (42.1–77.1)	92.1 (83.6–97.0)	0.860 (0.786–0.935)	0.678 (0.516–0.800)
	Test	64.3 (35.0–87.2)	81.2 (63.6–92.8)	0.779 (0.635–0.923)	0.621 (0.375–0.800)

Overall, the LR model incorporating radiomics information alone showed a significantly higher test-set AUC than the limited clinical model (DeLong *p* = 0.049), whereas the addition of clinical parameters to radiomics features did not further improve test-set AUC (DeLong *p* = 0.929).

In the *post hoc* restricted repeated resampling analysis, 29 of 100 repetitions fulfilled the predefined feature-set size criterion of 3–9 selected features. The remaining 71 repetitions were excluded from performance estimation because LASSO selected either too few or too many features for a comparable parsimonious model, indicating limited stability of the feature-selection pipeline in this small dataset. Among the included repetitions, median test-set AUC was 0.663 (IQR, 0.623–0.699), with median sensitivity of 0.286 (IQR, 0.214–0.357) and median specificity of 0.844 (IQR, 0.781–0.875). Median F1 score was 0.348 (IQR, 0.261–0.417). Arterial-venous difference features accounted for 60.9% of selected features. The ten most frequently selected features and their selection frequencies are provided in Table 6. Overall, these findings indicate limited stability of feature selection and model performance, while still supporting the hypothesis that phase-dependent radiomics information may contain relevant signal.

**Table 6:** The table shows the ten most frequently selected features among the 29 repetitions that fulfilled the predefined feature-count criterion of 3–9 LASSO-selected features. Percentages refer to these 29 included repetitions. Difference features represent arterial-venous difference features computed at the feature level.

Feature	Feature Type	Selection frequency, *n* (%)
diff_wavelet_HLL_firstorder_Skewness	Difference	19 (65.5%)
diff_wavelet_LLL_glszm_LargeAreaHighGrayLevelEmphasis	Difference	19 (65.5%)
arterial_original_glcm_MCC	Arterial	15 (51.7%)
diff_wavelet_HLL_glrlm_RunEntropy	Difference	10 (34.5%)
diff_wavelet_LLL_glszm_GrayLevelNonUniformity	Difference	8 (27.6%)
diff_wavelet_LLL_firstorder_10Percentile	Difference	7 (24.1%)
arterial_original_gldm_DependenceEntropy	Arterial	4 (13.8%)
arterial_wavelet_LLL_firstorder_Skewness	Arterial	4 (13.8%)
diff_original_shape_SurfaceVolumeRatio	Difference	3 (10.3%)
diff_wavelet_LLL_gldm_DependenceNonUniformity	Difference	3 (10.3%)

## 4 Discussion

Post-CA AKI is a frequent manifestation of the post-CA syndrome and contributes substantially to morbidity and mortality among survivors of OHCA (2, 5). Its pathophysiology is multifactorial. Tissue ischemia during the low-flow/no-flow period is followed by reperfusion, triggering a sepsis-like systemic response with distant organ damage (23) and post-resuscitation circulatory shock leads to sustained renal hypoperfusion which is a predictor for incidence and severity of post-CA AKI (4, 24–26). Clinically, post-arrest AKI is independently associated with prolonged hospitalization, worse neurological outcomes, and higher mortality (2, 4, 5, 27). Yet early risk stratification remains challenging because conventional predictors—vital signs, routine laboratory tests, and serum creatinine—are confounded by dynamic resuscitation physiology and often lag behind actual parenchymal injury (5). Reliable early prediction is therefore desirable to enable intensified monitoring, tailored hemodynamic management, avoidance of nephrotoxins, and timely nephrology involvement, with the potential to mitigate complications and reduce resource use.

This retrospective study included patients with OHCA who underwent standardized dual-phase contrast-enhanced CT at hospital admission. AKI occurred in 30.3% of cases, which is slightly lower than contemporary meta-analytic estimates in OHCA survivors (≈40% 2). The lower incidence is possibly explained by the exclusion of patients who died within the first five days after admission. A five-day observation window was chosen to distinguish patients who truly developed cardiac-arrest-related AKI from those who might have died before biochemical injury could manifest. A shorter window could have misclassified some high-risk patients as “no AKI” simply because death occurred prior to creatinine rise. Consistent with this rationale, the temporal creatinine trajectory in the cohort showed a peak around post-arrest day 3, aligning with published observations (5).

Initial kidney masks were generated automatically on arterial and venous phase images using TotalSegmentator and subsequently visually reviewed. Radiomics features were extracted for each phase, and difference features (arterial minus venous) were calculated to capture phase-dependent enhancement dynamics as a possible indicator of parenchymal perfusion and alterations. Before modeling, non-reproducible features were removed and the remaining features were normalized. To obtain a performance estimate, the dataset was split *a priori* into stratified training and independent test cohorts. Feature selection involved the identification of relevant features, exclusion of redundant features (mRMR), followed by further reduction of the feature count through LASSO regression to an appropriate feature-set size to minimize the risk of overfitting, all performed strictly within the training cohort to maximize robustness and generalizability of the final model.

The results suggest that radiomics-based risk stratification of AKI after OHCA may be feasible, with all three classifiers achieving moderate to promising discriminatory performance in the primary analysis. However, the performance estimates should be interpreted cautiously because the confidence intervals were wide, particularly in the internal test cohort, indicating statistical uncertainty in this relatively small exploratory cohort. In the internal test cohort, LR showed the most robust performance in the primary split (AUC 0.783), while SVM and kNN yielded comparable but slightly less consistent results (AUC 0.757 and 0.778, respectively). Importantly, the *post hoc* restricted repeated resampling analysis further demonstrated limited stability of both feature selection and model performance. Only 29 of 100 repetitions fulfilled the predefined criterion of 3–9 LASSO-selected features, indicating that the feature-selection pipeline did not consistently yield a parsimonious feature set of comparable size across different data partitions and needs to be further optimized. Among these included repetitions, median test-set AUC was only 0.663 (IQR, 0.623–0.699), which was lower than the AUC observed in the primary single-split analysis. These findings suggest that the primary performance estimate may be optimistic and should not be interpreted as evidence of a stable predictive model. Nevertheless, the selected predictors in the primary model were predominantly difference features, which also accounted for the majority of selected features in the repeated resampling analysis. This supports the hypothesis that phase-dependent enhancement dynamics, possibly reflecting perfusion alterations, may contain information relevant to early AKI risk stratification after CA, aligning with post-CA AKI pathophysiology (5). The similarity of performance across model families in the primary analysis suggests that the underlying feature-outcome relationship may not be highly complex and can potentially be captured with a relatively simple, largely linear model. LR has the advantage of being a relatively simple and interpretable model that may generalize better when the number of features is low. Consistent with this expectation, SVM and kNN performed very well in training but did not translate their apparent advantage to the internal test set, which is compatible with residual overfitting.

Notably, the limited clinical model based on pre-/intra-arrest variables alone had limited predictive ability, whereas the combination of clinical data and radiomics features did not substantially outperform radiomics alone. These findings suggest that radiomics may provide complementary information beyond the selected admission clinical variables. However, the clinical model was intentionally restricted to variables consistently available at admission and should not be interpreted as representing the full predictive potential of comprehensive clinical models.

Despite the promising discriminatory performance observed in the primary split, overall model performance remains suboptimal and would require substantial improvement before any clinical use could be considered. This is further supported by the repeated resampling analysis, which showed only moderate median discriminatory performance. In particular, sensitivity at the applied classification thresholds was modest across models and remained low in the repeated resampling analysis. This is clinically relevant because an AKI risk-stratification tool would need to reliably identify high-risk patients in order to support intensified monitoring, nephroprotective strategies, or early nephrology involvement. Therefore, the present models should be interpreted as exploratory and hypothesis-generating and not as stand-alone screening tools.

Our findings align with a growing body of literature suggesting that imaging markers and radiomics can provide clinically meaningful information for characterizing and prognosing renal disease. In chronic kidney disease contexts, Zhang et al. demonstrated that whole-kidney radiomics from non-contrast CT can identify early stages of chronic kidney disease (19), while Białek et al. reported that non-enhanced CT radiomics outperforms simple volumetric measures for detecting impaired renal function, underscoring the added value beyond morphology (17). Complementing these diagnostic studies, Choi et al. linked CT-derived radiomic signatures to biopsy-proven chronicity, providing biological plausibility for texture features as markers of parenchymal injury (28). In critical illness, Boutin et al. offered early evidence that abdominal CT radiomics can predict AKI in sepsis (13), and more recently Han et al. introduced a multimodal framework, which includes CT radiomics, for prediction of sepsis-associated AKI, where radiomics contributed useful information (16). Notably, Han et al. found clinical variables to be stronger predictors than radiomics. This observation stands in contrast with our study and is plausibly explained by timing and context. In the very early post-CA window, conventional clinical parameters may be less informative because hemodynamics is highly unstable and shock severity is not fully captured by the used variables available at admission, whereas dual-phase radiomics may more directly reflect perfusion characteristics relevant to imminent renal injury.

This work has several important limitations. First, the retrospective, single-center design limits causal inference and generalizability. Case identification, data completeness, and management practices may introduce selection bias and information bias. Furthermore, exclusion of patients who died within five days after admission may have introduced survivorship bias, as patients dying early after OHCA likely represent a particularly high-risk subgroup for multiorgan dysfunction, including AKI. Therefore, the findings apply primarily to patients surviving long enough for creatinine-based AKI assessment and should not be generalized to the entire OHCA population without further validation. In addition, the sample size was limited for radiomics-based machine learning modeling, particularly with only 47 AKI events. Although model complexity was reduced by reproducibility filtering, mRMR-based feature preselection, and LASSO regression, residual overfitting cannot be excluded. This is especially relevant given the small study cohort. The *post hoc* restricted repeated resampling analysis further demonstrated limited feature-selection stability, as only 29 of 100 repetitions yielded a predefined feature-set size of 3–9 LASSO-selected features suitable for performance estimation. These findings suggest that the applied feature-selection pipeline, although useful for dimensionality reduction in the present exploratory setting, had limited robustness and may not be optimal for generalizable model development in small cohorts. Future studies should therefore further optimize and validate the feature-selection strategy, ideally within larger multicenter datasets. Furthermore, feature scaling was performed as an unsupervised preprocessing step on the full dataset, which may represent a minor methodological limitation despite not using AKI outcome information. Despite separation of all outcome-related feature selection and model-training steps from the internal test cohort, findings should be validated with external, multicenter cohorts. Second, only one scanner and a single, standardized dual-phase CT protocol were used for analysis. Radiomics features can vary with acquisition and reconstruction parameters, as well as contrast timing and dosing. Different protocols therefore might lead to varying feature distribution and model performance. Moreover, arterial and venous phase images were reconstructed with different slice thicknesses and kernels according to the standardized institutional protocol. Because this protocol was applied consistently across the entire cohort, these technical differences were systematic rather than patient-specific. Nevertheless, arterial-venous difference features may still partly incorporate reconstruction-related effects rather than purely biological enhancement patterns. Although all kidney segmentation masks underwent visual quality control, no formal local segmentation-performance evaluation against fully manual ground-truth annotations was performed. Therefore, segmentation-related uncertainty cannot be fully excluded, although major segmentation errors are considered unlikely given the use of an established automated segmentation tool, visual quality control, and the anatomically well-defined target structure. For the limited clinical model, the clinical covariates were intentionally limited to variables available at admission and previously described as predictors for post-CA AKI. Important risk markers such as baseline eGFR, comorbidity burden, lactate, vasopressor dose, urine output, and detailed hemodynamic indices were not incorporated, which may underestimate the potential of clinical models. Future, preferably multicenter studies should move beyond discrimination and systematically evaluate model calibration and clinical utility, alongside threshold-specific performance, to support potential clinical translation.

Notwithstanding these limitations, this study provides initial exploratory evidence that radiomics features extracted from contrast-enhanced CT may contain information relevant to AKI risk stratification after OHCA, with arterial-venous difference features appearing particularly informative. Importantly, the study was based on a clinically relevant OHCA cohort in which CT imaging was not selectively performed for a specific clinical indication, but was acquired according to a standardized institutional post-cardiac arrest imaging protocol for all patients stable enough to undergo CT. This setting represents a particular strength, as such systematically imaged post-cardiac arrest cohorts are uncommon and may reduce imaging-related selection bias. Given the limited stability of feature selection and model performance, these findings should nevertheless be considered hypothesis-generating and require substantial methodological refinement and external validation before any clinical application can be considered.

## 5 Conclusion

Dual-phase contrast-enhanced CT radiomics may provide additional information for the early prediction of AKI after OHCA. In this exploratory single-center study, features capturing differences between arterial and venous phases appeared particularly informative, suggesting that phase-dependent renal enhancement patterns could reflect early pathophysiological alterations relevant to subsequent kidney injury. However, the limited stability of feature selection and model performance indicates that the present findings should be interpreted as hypothesis-generating rather than as evidence of a robust predictive model. Further methodological refinement and validation in prospective multicenter cohorts are essential before clinical application can be considered.

## Data Availability

The datasets presented in this article are not publicly available due to privacy and ethical restrictions. Data may be made available by the corresponding author upon reasonable request. Requests to access the datasets should be directed to michael.scheschenja@med.uni-marburg.de.
