# Supplementary material for: Dual-phase CT radiomics for acute kidney injury prediction after out-of-hospital cardiac arrest
Source: Front Radiol. 2026 Jun 26;6:1875412. doi: 10.3389/fradi.2026.1875412 (PMC13350171; doi:10.3389/fradi.2026.1875412)
Supplement: Supplementary file 1 [file Datasheet1.pdf]

# METRICS Tool v1.0

**Please fill out all conditions first for relevant sections and then all active items to calculate METRICS score.**

**Please note that default option is "No".**

? Stands for explanation of items and conditions.

C Stands for conditional items or sections.

| Items/Conditions | Definitions | Weights | Options |
|---|---|---|---|
| **Study Design** | | | |
| Item#1 | ? Adherence to radiomics and/or machine learning-specific checklists or guidelines | 0.0368 | ◉ Yes ○ No |
| Item#2 | ? Eligibility criteria that describe a representative study population | 0.0735 | ◉ Yes ○ No |
| Item#3 | ? High-quality reference standard with a clear definition | 0.0919 | ◉ Yes ○ No |
| **Imaging Data** | | | |
| Item#4 | ? Multi-center | 0.0438 | ○ Yes ◉ No |
| Item#5 | ? Clinical translatability of the imaging data source for radiomics analysis | 0.0292 | ◉ Yes ○ No |
| Item#6 | ? Imaging protocol with acquisition parameters | 0.0438 | ◉ Yes ○ No |
| Item#7 | ? The interval between imaging used and reference standard | 0.0292 | ◉ Yes ○ No |
| **Segmentation** C | | | |
| Condition#1 | ? Does the study include segmentation? | | ◉ Yes ○ No |
| Condition#2 | ? Does the study include fully automated segmentation? | | ○ Yes ◉ No |
| Item#8 | ? Transparent description of segmentation methodology | 0.0337 | ◉ Yes ○ No |
| Item#9 | ? Formal evaluation of fully automated segmentation C | 0.0225 | ○ Yes ○ No |
| Item#10 | ? Test set segmentation masks produced by a single reader or automated tool | 0.0112 | ◉ Yes ○ No |
| **Image Processing and Feature Extraction** | | | |
| Condition#3 | ? Does the study include hand-crafted feature extraction? | | ◉ Yes ○ No |
| Item#11 | ? Appropriate use of image preprocessing techniques with transparent description | 0.0622 | ◉ Yes ○ No |
| Item#12 | ? Use of standardized feature extraction software C | 0.0311 | ◉ Yes ○ No |
| Item#13 | ? Transparent reporting of feature extraction parameters, otherwise providing a default configuration statement | 0.0415 | ◉ Yes ○ No |
| **Feature Processing** | | | |
| Condition#4 | ? Does the study include tabular data? | | ◉ Yes ○ No |
| Condition#5 | ? Does the study include end-to-end deep learning? | | ○ Yes ◉ No |
| Item#14 | ? Removal of non-robust features C | 0.0200 | ◉ Yes ○ No |
| Item#15 | ? Removal of redundant features C | 0.0200 | ◉ Yes ○ No |
| Item#16 | ? Appropriateness of dimensionality compared to data size C | 0.0300 | ◉ Yes ○ No |
| Item#17 | ? Robustness assessment of end-to-end deep learning pipelines C | 0.0200 | ○ Yes ○ No |
| **Preparation for Modeling** | | | |
| Item#18 | ? Proper data partitioning process | 0.0599 | ◉ Yes ○ No |
| Item#19 | ? Handling of confounding factors | 0.0300 | ◉ Yes ○ No |
| **Metrics and Comparison** | | | |
| Item#20 | ? Use of appropriate performance evaluation metrics for task | 0.0352 | ◉ Yes ○ No |
| Item#21 | ? Consideration of uncertainty | 0.0234 | ◉ Yes ○ No |
| Item#22 | ? Calibration assessment | 0.0176 | ○ Yes ◉ No |
| Item#23 | ? Use of uni-parametric imaging or proof of its inferiority | 0.0117 | ○ Yes ◉ No |

| | | | | |
|---|---|---|---|---|
| Item#24 | ? | Comparison with a non-radiomic approach or proof of added clinical value | 0.0293 | (●) Yes ( ) No |
| Item#25 | ? | Comparison with simple or classical statistical models | 0.0176 | ( ) Yes (●) No |
| **Testing** | | | | |
| Item#26 | ? | Internal testing | 0.0375 | (●) Yes ( ) No |
| Item#27 | ? | External testing | 0.0749 | ( ) Yes (●) No |
| **Open Science** | | | | |
| Item#28 | ? | Data availability | 0.0075 | ( ) Yes (●) No |
| Item#29 | ? | Code availability | 0.0075 | ( ) Yes (●) No |
| Item#30 | ? | Model availability | 0.0075 | ( ) Yes (●) No |
| | | | **Total METRICS score:** | **80.4%** |
| | | | ? **Quality category:** | **Excellent** |
| | | | ? **Publication ID:** | CA-AKI |

**If you publish any work which uses this tool, please cite the following publication:**

Kocak B, Akinci D'Antonoli T, Mercaldo N, et al. METhodological RadiomICs Score (METRICS): a quality scoring tool for radiomics research endorsed by EuSoMII. Insights Imaging. 2024;15(1):8. Published 2024 Jan 17. doi:10.1186/s13244-023-01572-w
