# Supplementary material for: Dual-phase CT radiomics for acute kidney injury prediction after out-of-hospital cardiac arrest
Source: Front Radiol. 2026 Jun 26;6:1875412. doi: 10.3389/fradi.2026.1875412 (PMC13350171; doi:10.3389/fradi.2026.1875412)
Supplement: Supplementary file 3 [file Table1.docx]

| Feature | AKI (n = 14) | No AKI (n = 32) |
| --- | --- | --- |
| diff_wavelet_HLL_firstorder_Skewness (SD) | 0.069 (0.084) | -0.021 (0.152) |
| diff_wavelet_LLL_firstorder_10Percentile (SD) | 0.088 (0.090) | 0.106 (0.074) |
| diff_wavelet_LLL_gldm_LargeDependenceHighGrayLevelEmphasis (SD) | -0.015 (0.080) | -0.056 (0.187) |
| diff_wavelet_LLL_glszm_GrayLevelNonUniformity (SD) | -0.205 (0.100) | -0.074 (0.204) |
| diff_wavelet_LLL_glszm_LargeAreaHighGrayLevelEmphasis (SD) | 0.120 (0.122) | -0.041 (0.200) |
| feature_A_original_glcm_MCC (SD) | 0.483 (0.226) | 0.643 (0.214) |
| feature_A_wavelet_HLL_glrlm_RunEntropy (SD) | 0.265 (0.170) | 0.387 (0.201) |

## Feature characteristics (final selected radiomics; normalized (SD)) – Test cohort by AKI status

| Feature | AKI (n = 33) | No AKI (n = 76) |
| --- | --- | --- |
| diff_wavelet_HLL_firstorder_Skewness (SD) | 0.040 (0.077) | -0.019 (0.100) |
| diff_wavelet_LLL_firstorder_10Percentile (SD) | 0.147 (0.084) | 0.107 (0.089) |
| diff_wavelet_LLL_gldm_LargeDependenceHighGrayLevelEmphasis (SD) | 0.029 (0.115) | -0.073 (0.188) |
| diff_wavelet_LLL_glszm_GrayLevelNonUniformity (SD) | -0.153 (0.116) | -0.068 (0.149) |
| diff_wavelet_LLL_glszm_LargeAreaHighGrayLevelEmphasis (SD) | 0.069 (0.172) | -0.033 (0.141) |
| feature_A_original_glcm_MCC (SD) | 0.574 (0.195) | 0.687 (0.158) |
| feature_A_wavelet_HLL_glrlm_RunEntropy (SD) | 0.342 (0.203) | 0.479 (0.204) |

## Feature characteristics (final selected radiomics; normalized (SD)) – Training cohort by AKI status

| Characteristic | AKI (n = 14) | No AKI (n = 32) |
| --- | --- | --- |
| Age (years (SD)) | 71.1 (10.5) | 71.7 (12.3) |
| Time to ROSC (min (SD)) | 17.7 (10.6) | 21.0 (13.3) |
| Creatinine at admission (mg/dL (SD)) | 1.29 (0.45) | 1.24 (0.29) |
| Sex: m (n (%)) | 10 (71.4%) | 24 (75.0%) |
| Sex: w (n (%)) | 4 (28.6%) | 8 (25.0%) |
| Initial rhythm: Asystole (n (%)) | 4 (28.6%) | 7 (21.9%) |
| Initial rhythm: Ventricular fibrillation/flutter (n (%)) | 5 (35.7%) | 17 (53.1%) |
| Initial rhythm: Pulseless electrical activity (n (%)) | 5 (35.7%) | 8 (25.0%) |

## Demographic characteristics – Test cohort by AKI status

| Characteristic | AKI (n = 33) | No AKI (n = 76) |
| --- | --- | --- |
| Age (years (SD)) | 70.1 (15.3) | 69.6 (13.4) |
| Time to ROSC (min (SD)) | 21.7 (11.7) | 17.5 (10.6) |
| Creatinine at admission (mg/dL (SD)) | 1.31 (0.34) | 1.15 (0.30) |
| Sex: m (n (%)) | 24 (72.7%) | 57 (75.0%) |
| Sex: w (n (%)) | 9 (27.3%) | 19 (25.0%) |
| Initial rhythm: Asystole (n (%)) | 12 (36.4%) | 16 (21.1%) |
| Initial rhythm: Ventricular fibrillation/flutter (n (%)) | 13 (39.4%) | 44 (57.9%) |
| Initial rhythm: Pulseless electrical activity (n (%)) | 8 (24.2%) | 16 (21.1%) |

## Demographic characteristics – Training cohort by AKI status
